# Treatment of Adolescent Alcohol Abuse and Dependence

**Published:** 1994

**Authors:** Oscar G. Bukstein

**Affiliations:** Oscar G. Bukstein, M.P. H., M. D., is an assistant professor of child psychiatry at the University of Pittsburgh School of Medicine and the medical director of the Child and Parent Behavior Clinic at Western Psychiatric Institute and Clinic, Pittsburgh, Pennsylvania

## Abstract

Most adolescents under the legal drinking age consume alcohol. For many of them, their drinking patterns evolve into alcohol abuse and dependence. Treatment of these adolescents must take into consideration their physical, psychological, and social development. Therefore, different treatment approaches may be needed for adolescents than for adults.

Alcohol abuse and alcohol dependence^1^ are not only adult problems. They also affect a significant portion of adolescents between the ages of 12 and 18, even though the purchase and public use of alcohol are illegal for them. Diagnosis and treatment of these disorders are important because alcohol-related problems can have an enormous impact on the adolescents’ future. Early alcohol abuse can lead to social, family, and developmental problems. Other potential negative consequences include accidents (e.g., motor vehicle crashes, falls, and drownings), aggressive behavior, and suicide (National Institute on Alcohol Abuse and Alcoholism [NIAAA] 1990). Alcohol use also is associated with other risk-taking behaviors, such as smoking, delinquency, use of illicit drugs, and precocious sexual behavior (Donovan and Jessor 1978; Braucht 1982). Furthermore, many alcohol-abusing adolescents become alcohol-abusing adults (Temple and Fillmore 1986).

This article reviews the prevalence of alcohol abuse and dependence among adolescents and examines the characteristics of abuse and the unique needs of adolescents that must be considered when devising treatment plans for these patients. The article also discusses the contents and effectiveness of current treatment approaches and addresses future directions for adolescent alcoholism treatment research.

## Prevalence of Adolescent Alcohol Use and Abuse

Alcohol use is widespread among adolescents. In an annual survey of high school students, almost 90 percent of the seniors reported having at least tried alcohol (Johnston et al. 1993). The same survey found that almost one-third of high school seniors had five or more drinks on at least one occasion in the previous 2 weeks.

How many adolescents have serious alcohol-related problems? Prevalence rates of alcohol abuse and dependence as determined in community studies vary widely. Kashani and colleagues (1987) found that 5.3 percent of the 15-year-olds studied fulfilled the criteria for alcohol abuse or dependence listed in the *Diagnostic and Statistical Manual of Mental Disorders, Third Edition* (DSM–III). Another study reported a lifetime diagnosis of alcohol abuse or dependence as defined in the 1987 *Diagnostic and Statistical Manual of Mental Disorders, Third Edition, Revised* (DSM–III–R) in 32.4 percent of adolescents ages 17 to 19 (Reinherz et al. 1993). In addition, a survey of students in grades 7 through 12 identified 28 percent of male and 16 percent of female adolescents as problem drinkers (Donovan and Jessor 1978).

These studies, however, must be interpreted with caution. The diagnoses in these surveys were based on an arbitrary definition of problem use or on the DSM criteria, which have been established for adults. The validity of these definitions for a diagnosis in adolescents remains controversial (Bukstein and Kaminer 1994). For example, the DSM–III-based diagnosis of alcohol dependence relies heavily on the presence of specific withdrawal symptoms, which rarely are found in adolescents. Moreover, establishing a diagnosis of alcohol abuse on the grounds of medical or social problems or functional impairment may be doubtful, because these symptoms also could be caused by other problem behaviors, psychiatric disorders, or adverse environmental circumstances that often are found in alcohol-abusing adolescents.

## Characteristics of Adolescent Alcohol Abuse

Alcohol-abusing adolescents may require not only a specific, age-appropriate set of diagnostic criteria but also treatment approaches different from those for adults. Adolescent drinking behavior has special characteristics that set it apart from adult drinking behavior and that should be recognized if treatment is to be effective.

One characteristic of adolescent alcohol use is the variability of drinking patterns. For most people, these patterns change between adolescence and young adulthood and then stabilize (NIAAA 1990). Temple and Fillmore (1986), for example, found that only one-half of the heavier drinkers at age 18 continued the same drinking pattern 12 years later. Therefore, alcohol abuse in many adolescents may reflect a developmental phase that ceases over time and requires little or no intervention. To date, no objective criteria exist to determine which adolescents need or can benefit from treatment.

Compared with adults, adolescents are more likely to use and abuse other drugs in addition to alcohol, potentially complicating treatment (Bukstein and Van Hasselt 1993). The reasons for multiple drug use among adolescents are not completely clear, but temperament or personality traits, such as disinhibition or sensation-seeking behavior, may encourage multiple drug use.

However, adolescents are less likely than adults to suffer from alcohol-related chronic medical problems and psychological withdrawal symptoms (Bukstein and Van Hasselt 1993). This may be a result of their relatively short drinking career, and it is possible that overt health problems will develop after significant alcohol consumption over a longer period of time.

Adolescence frequently marks the onset not only of alcohol use and abuse but also of psychiatric disorders, such as conduct disorder (i.e., a pattern of antisocial behaviors, for example, lying, stealing, fighting, and truancy), major depression or bipolar disorder (i.e., alternating episodes of depression and mania), and anxiety disorders (Bukstein et al. 1989). These coexisting psychiatric disorders may increase adolescents’ vulnerability to alcohol abuse or may be the consequence of alcohol abuse (Bukstein et al. 1989). Attention to psychiatric disorders, therefore, is crucial in the treatment of alcohol-abusing adolescents.

## The Unique Needs of Adolescents in Treatment

In the past, adolescents generally received treatment originally designed for adults. Only in 1974 did the first facility specifically for adolescent substance abusers open as an inpatient program at St. Mary’s Hospital in Minneapolis, MN (Wheeler and Malmquist 1987). Based on clinical observation, treatment providers since then have recognized increasingly that adolescents are exposed to specific influences and have special needs (see textbox) during the transition from childhood to adulthood. Consequently, they require treatment approaches that understand and address their position and roles in family and society, their cognitive and social development, the environmental influences on their behavior, and their educational requirements.

**Developmental Characteristics of Adolescents Relevant to Treatment of Alcohol Abuse and Dependence**

Dependent position in family and society

Limits imposed by physical, social, and cognitive development

Greater influence by peers and popular culture

Need for educational or vocational training

Frequent coexisting psychiatric disorders

Frequent multiple drug abuse

The position as a dependent family member is a salient aspect of an adolescent’s life and can have important implications for the development and treatment of alcohol abuse. The family should provide support, consistent behavioral limits, and models of appropriate behavior (including drinking behavior). Deficits in these domains, including parental alcohol or other drug abuse, are seen as risk factors for adolescent alcohol abuse or dependence (Kumpfer 1989). These deficits also can influence treatment outcome by hampering the family’s ability to encourage the adolescent’s entry into treatment, to participate in treatment, and to provide adequate structure and guidance afterward.

Adolescents undergo not only physical maturation but also cognitive maturation (e.g., development of abstract thinking) and social maturation (e.g., development of values to guide behavior). In addition, they must develop a separate identity and independence from their parents and build appropriate societal and individual relationships to prepare for adult life with a job, marriage, and family. In this transition period, adolescents experiment with a wide variety of attitudes and behaviors. Successful treatment programs should provide guidance and support through these important stages in adolescent development.

Environmental influences also contribute significantly to the developing patterns of alcohol use in adolescents. Peer behavior (including alcohol use) and attitudes, media messages, community values, and social support systems all help shape adolescents’ beliefs about alcohol and their drinking patterns (Kumpfer 1989). Therefore, it is important that treatment interventions foster the development of effective social skills to enhance resistance to negative peer influences.

Finally, adolescents’ ongoing education or vocational training also must be considered when treatment programs are developed. To prepare for adult life, adolescents must achieve at least a basic level of academic skills, and treatment programs therefore should require or strongly encourage completion of a secondary education.

## Current Treatment Approaches

In a 1991 survey, adolescents under age 18 constituted about 6 percent of the patients in alcoholism and drug abuse treatment units (U.S. Department of Health and Human Services, Substance Abuse and Mental Health Services Administration 1993). About 35 percent of these clients were treated for alcoholism and about 40 percent for alcoholism and other drug abuse. The same survey showed that 3,299 treatment units offered specialized programs for youth. These programs offer a variety of inpatient and outpatient treatment modalities (table 1).

The predominant inpatient approach remains the Minnesota model, a group-oriented 28-day program that is based on a 12-step program of recovery (Wheeler and Malmquist 1987). Counseling, attendance at group meetings, information about alcoholism and related problems, family therapy with individual families and multifamily groups, and the use of workbooks are common components of this and related programs. Aftercare following program completion varies in content and often centers on attendance at community Alcoholics Anonymous (AA) meetings. Although most meetings include participants who represent a wide age range, some communities have AA groups that are attended predominantly by youth and deal with issues more relevant to adolescents.

Residential programs, such as therapeutic communities or halfway houses, usually have a similar orientation to that of inpatient programs but offer long-term treatment. Also, they often are designed to serve youth with more severe drug abuse or coexisting psychosocial problems (Bukstein and Van Hasselt 1993).

Outpatient treatment modalities, which also are based mainly on 12-step programs, range from very intensive day programs and partial hospitalization to regular or occasional sessions with an individual therapist. The primary advantage of outpatient treatment is that it is often less disruptive to the adolescent, who can participate in treatment while remaining in the community. However, this also can be a disadvantage because the adolescent still is exposed to circumstances, stressors, and problems that may have contributed to the alcohol problems. In addition, the treatment provider has less control over the adolescent’s behavior outside treatment hours.

## Variations in Current Treatment Approaches

As with alcohol and other drug abuse treatment for adults, treatment approaches for adolescents currently are undergoing rapid changes, for example, by reducing their costs and emphasizing less restrictive treatment modalities. Many traditional 28- or 35-day inpatient programs are decreasing their length of stay and are expanding outpatient and partial hospitalization services. Some programs that are unable to achieve this transition are closing or reducing their capacity (Spicer 1993). It still is uncertain if and how these trends will affect treatment.

At the same time, there is a growing interest in alternative approaches (Bukstein and Van Hasselt 1993). Many programs have begun to incorporate a variety of family or behavioral treatments, health services, vocational and educational services, and recreational activities in addition to 12-step principles. Other initiatives treat alcohol-abusing youth as part of a comprehensive system of services for hard-to-reach, multiproblem adolescents and their families (Henggeler 1991). In these programs, case managers and multidisciplinary teams from different social service agencies and treatment programs coordinate services and care. Also, increasing emphasis is placed on providing help to the adolescents in their own community and in as “normal” a setting as possible.

Some treatment programs are paying more attention to subpopulations of adolescent patients. They specifically address adolescents with coexisting psychiatric disorders, homeless youth, inner-city youth, youth from specific racial or ethnic groups, and adolescents in the juvenile justice system (Bukstein and Van Hasselt 1993).

Especially for adolescents with coexisting psychiatric disorders, psychological and behavioral interventions appear to be used increasingly (Bukstein and Van Hasselt 1993). Many of these approaches originally were developed for prevention programs and have been adapted for treatment. Examples of such interventions include improvement of social skills (e.g., communication and assertiveness skills), problem-solving skills, anger control training, and family counseling (Bukstein and Van Hasselt 1993). The latter may contain contingency contracting, an explicit agreement between the parent(s) and the adolescent about the rules and expectations of the household. Contracts specify the consequences if the rules are violated and the rewards if they are obeyed.

The increased recognition of coexisting psychiatric disorders also has prompted more aggressive efforts to use pharmacologic treatment with these patients. For adolescents as well as adults, the use of antidepressants, lithium, and other agents is becoming more accepted. For adolescent alcohol abusers who have attention-deficit hyperactivity disorder, however, treatment with stimulants remains controversial because these medications also have a potential for abuse (Bukstein and Van Hasselt 1993).

## Evidence of Treatment Efficacy

Although different treatment approaches are being used for adolescents with alcohol abuse or dependence, rigorous evaluation of their effectiveness is scarce. Many studies were flawed by various methodological problems, including poor pre-assessment measures, lack of clear definitions and measures of treatment success or relapse, and insufficient followup procedures. The available studies generally suggest beneficial effects of treatment for adolescents but do not establish the superiority of any specific approach (Catalano et al. 1990–1991). Two studies that evaluated several adolescent treatment options, the Drug Abuse Reporting Program and a study for the Pennsylvania substance abuse system, found that adolescents in outpatient programs for alcohol abuse, but not those in inpatient programs, reported a moderate reduction in alcohol use (Sells and Simpson 1979; Rush 1979). In contrast, in the Treatment Outcome Prospective Study (TOPS), Hubbard and colleagues (1985) noted significant improvements of alcohol-related problems in both inpatients and outpatients. This study showed that patients in residential settings reported a greater reduction in heavy alcohol use than did outpatients.

In a study of adolescent treatment in an Alcoholics Anonymous-Narcotics Anonymous (AA-NA) model inpatient program, both completers and noncompleters of the program had reduced alcohol and other drug use after treatment (Alford et al. 1991), but completers showed lower relapse rates 6, 12, and 24 months after discharge than did noncompleters. Among males in both groups, however, relapse rates had exceeded 60 percent by the 1-year followup. In contrast, females only reported relapse rates of 30 percent at the 1-year followup and 39 percent at the 2-year followup. And even among the adolescents of both genders who had relapsed, 17 percent reported improvement in their social behavior at the 2-year followup.

All these studies of adolescent alcoholism treatment, however, are limited by the absence of control groups or treatments, the lack of control over the high variability of treatment content among programs, and the absence of data on the treatment effects on areas of psychosocial functioning.

As indicated by the AA-NA study, many adolescents relapse into alcohol abuse patterns within a relatively short time after treatment completion. In a recent review of adolescent treatment research, Catalano and colleagues (1990–1991) identified factors influencing treatment outcome and relapse risk. For example, earlier and heavier alcohol abuse, multiple drug abuse, deviant behavior, and criminal involvement were associated with noncompletion of treatment or higher relapse rates. Posttreatment predictors of relapse included thoughts and cravings for alcohol, little involvement in school or work, and unsatisfactory leisure-time activities.

## What Makes Treatment Effective?

Despite the inconclusive results of the studies mentioned above, some general recommendations can be made for adolescent treatment modalities (Fleisch 1991). As with all treatment programs, the primary goal should be to achieve and maintain abstinence from alcohol and all illicit drugs. Treatment should improve the overall psychosocial functioning (e.g., educational, vocational, family, and interpersonal functioning) of the adolescent as well as the specific areas (e.g., problem-solving or anger control skills) that particularly may help the adolescent to avoid relapse.

Several specific treatment characteristics have been associated with improved abstinence and lower relapse rates (Fleisch 1991; Friedman and Beschner 1985) and can be used as guidelines for the treatment of adolescents with alcohol use problems:

- Treatment should be intensive and of sufficient duration to achieve changes in attitude and behavior. What constitutes “sufficient” may depend on the treatment modality and the needs of the individual patient.
- Treatment should be as comprehensive as possible and target multiple domains of the adolescents’ lives. These include coexisting psychiatric disorders; vocational or educational needs; recreational activities; birth control services; education about alcohol and other drug abuse; and information about relevant medical issues, such as HIV infection and AIDS.
- Treatment should be sensitive to the cultural and socioeconomic realities of the adolescents, their families, and their environments. This can be achieved by having a staff that represents the racial and ethnic variety of the adolescents in treatment and by assisting families to obtain additional social services or financial resources.
- Treatment should encourage family involvement and improvement of communication among family members. The goal should be to enhance the ability of parents to provide proper guidance consistently for their children. To this end, addiction patterns in the parents also must be recognized and treated and their contribution to the adolescents’ addiction evaluated.
- Treatment programs should incorporate a wide range of social services, such as juvenile justice, child welfare, and recreational programs, to assist the adolescents and their families in developing an alcohol-and-other-drug-free lifestyle. Programs should require or strongly encourage adolescents’ attendance at self-support groups, such as AA, to provide a drug-free peer group.
- Aftercare is essential and should reinforce the changes that have been achieved during primary treatment.

## Future Directions for Adolescent Alcoholism Treatment Research

The available outcome studies have shown that a significant portion of alcohol-abusing adolescents do not respond to treatment with desired outcomes, such as abstinence, reduced drinking, or improvements in psychosocial functioning. In the TOPS study, between 29 and 40 percent of the patients (depending on the treatment modality) reported heavy alcohol use in the 12 months after treatment (Hubbard et al. 1985). To improve treatment outcome in adolescents who do not respond satisfactorily to current treatment approaches, several issues must be addressed in future research.

First, age-appropriate diagnostic criteria must be established to improve the identification of youth with alcohol abuse or dependence. Further research into the development of adolescent alcohol use is needed to recognize adolescents at risk for continued alcohol abuse and in need of treatment.

Second, treatment studies need more rigorous experimental design and methodology. This includes (1) comprehensive, standardized assessments before, during, and after treatment with, for example, assessment tools developed specifically for adolescents, such as the Problem-Oriented Screening Instrument for Teenagers (POSIT) (National Institute on Drug Abuse 1990) and the Personal Experience Inventory (Winters et al. 1993); (2) thorough inventories of treatment content (i.e., what kind of modalities are used and in what intensity); (3) manual-guided procedures for implementing specific interventions with specific contents; and (4) the use of appropriate controls.

Third, treatment outcomes must be evaluated more thoroughly, with careful followup for several years. Outcome studies not only should consider abstinence or relapse status but also should include all changes in drinking patterns and psychosocial functioning.

Fourth, nontraditional treatments, such as behavioral interventions (e.g., social and problem-solving training, cognitive therapy, and parent-management training to assist parents in setting behavioral limits) and medication treatment, should be evaluated further, especially in patients for whom more traditional approaches are not effective. As with adults, it is likely that certain types and intensities of treatment may be more suitable for certain adolescents. Patient-treatment matching, that is, designing an individualized treatment plan for each patient, will therefore be a critical part of treatment research.

Finally, treatment studies also should evaluate efficacy in specific populations of adolescents with regard to age, race, gender, ethnicity, socioeconomic status, and comorbid psychiatric status.

## Summary

By the time they graduate from high school, 90 percent of adolescents have tried alcoholic beverages, and many of them drink regularly. A significant portion of adolescents will suffer psychological and social problems because of abusive patterns of alcohol use and will require intervention. To treat them successfully, health care professionals must take into account the adolescents’ special developmental status and position in the family and society. Although many adolescents can be helped by current treatment programs, further research is needed to establish what kind of treatment, and at what intensity and duration, is necessary to reduce alcohol consumption and prevent relapse in patients currently unresponsive to treatment.

## Figures and Tables

**Table 1:** Current Approaches and Their Components for Alcoholism Treatment in Adolescents

Approach	Components and/or focus
12-Step Model	Group meetings, for example,
	Alcoholics Anonymous
	Group counseling
	Workbooks
	Family sessions or groups
Behavioral Intervention	Relapse prevention
	Development or improvement of refusal skills, social skills, and problem-solving skills
	Anger control training
	Leisure-time management
Family Therapy	
Behavioral	Parent management training
	Contingency contracting^1^
Strategic-structural	Restructure maladaptive patterns
Systemic	Address behavioral limit-setting and intergenerational conflicts
Contextual	Integration of emotional, behavioral, cognitive, and spiritual aspects of family
Mixed	A combination of various family therapy components
Educational and Vocational Assistance/Rehabilitation	Achieve basic level of academic skills and completion of a secondary education
Medications for Coexisting Psychiatric Disorders	
Depression and other mood disorders	Antidepressants and lithium
Attention-deficit hyperactivity disorder	Stimulants
Severe aggression	Antidepressants and lithium

1 Contingency contracting establishes rules between the parent(s) and adolescent and defines consequences for breaking those rules as well as rewards for obeying them.

## Footnotes

1 The terms “alcohol abuse” and “alcohol dependence” are based on the diagnostic criteria in the American Psychiatric Association’s *Diagnostic and Statistical Manual of Mental Disorders, Third Edition, Revised*.
